# Ecosystem function decays by fungal outbreaks in Antarctic microbial mats

**DOI:** 10.1038/srep22954

**Published:** 2016-03-14

**Authors:** David Velázquez, Alberto López-Bueno, Daniel Aguirre de Cárcer, Asunción de los Ríos, Antonio Alcamí, Antonio Quesada

**Affiliations:** 1 Departamento de Biología, Universidad Autónoma de Madrid, Madrid, Spain; 2 Centro de Biología Molecular Severo Ochoa, Consejo Superior de Investigaciones Científicas (CSIC)–Universidad Autónoma de Madrid, Madrid, Spain; 3 Museo Nacional de Ciencias Naturales–CSIC, Madrid, Spain

## Abstract

Antarctica harbours a remarkably diverse range of freshwater bodies and terrestrial ecosystems, where microbial mats are considered the most important systems in terms of biomass and metabolic capabilities. We describe the presence of lysis plaque-like macroscopic blighted patches within the predominant microbial mats on Livingston Island (Antarctic Peninsula). Those blighting circles are associated with decay in physiological traits as well as nitrogen depletion and changes in the spatial microstructure; these alterations were likely related to disruption of the biogeochemical gradients within the microbial ecosystem caused by an unusually high fungal abundance and consequent physical alterations. This phenomenon has been evidenced at a time of unprecedented rates of local warming in the Antarctic Peninsula area, and decay of these ecosystems is potentially stimulated by warmer temperatures.

Antarctica likely harbours the last undisrupted freshwater ecosystems on Earth^1^, and most of their biomass and productivity is housed within microbial mats^2^. These systems represent cyanobacteria-based communities growing on a biological porous matrix and vertically stratified^3^, where microorganisms orientate themselves in response to micrometer-scale physico-chemical gradients^4^,^5^. Small changes in physico-chemical zonation may modify species composition and metabolic pathways. Consequently, broader perturbations, such as climate change, have the potential to exacerbate changes of microbial ecosystems food-webs, as well as their role in the Antarctic ecological processes^6^.

During a 2001 field trip to Byers Peninsula (Antarctic Specially Protected Area No. 126; Livingston Island, Antarctic Peninsula)^7^ (Fig. 1), discoloured (blighted) circles were observed within the dominant microbial mats (Fig. 2A,B). These patches were widely distributed in the studied area and covered significant portions of the mat surface. Microbial mats extensively cover the seepage areas of the southwest part of Byers Peninsula. There, a total of 15 sites with multiple blighted circles were spotted along different transects (Fig. 1), indicating that this is a common feature in this location. The distribution of these patches showed no obvious relation with environmental characteristics such as water availability, terrain morphology or position within different drainage networks and only the most representatives 6 blighted and non-affected sites were sampled. Therefore, we monitored the diameter shifts of a number of these patches during 3 subsequent campaigns (2006–2010) and observed consistent and rapid growth in eight out of nine patches (Supplementary Table 1). We then compared non-affected and blighted mat samples from different locations and analysed their physical microstructure, physiology, and complete community composition of Eukarya, Bacteria, and also DNA and RNA viruses since they are known strong drivers of cyanobacteria-dominated ecosystems^8^.

## Results

### Lack of physical scaffolding

In addition to the observed macroscopic differences, low-temperature electron microscopy revealed a denser and less porous structure and absence of the largest pore sizes in the affected patches. Additionally, putative fungal structures were identified within large pores in the blighted mats (Fig. 2C).

### Reduction of C and N uptake ratios

The average photosynthetic incorporation rate was nearly two-fold lower (p-value < 0.05) in blighted patches (non-affected, 16.5 ± 6.1; blighted, 8.6 ± 1.1 μgC cm^−2^ h^−1^), which was accompanied by lower rates of N_2_ fixation (non-affected, 8.4 ± 4.1; blighted, 5.1 ± 1.0 nmol ethylene (g dry weight h)^−1^; p-value < 0.05). Consistent with these observations, the total C and N proportions changed on average 3.4-fold in non-affected mats compared to blighted patches (C, 5.40 ± 1.9% and 18.19 ± 2.9%; N, 1.7 ± 0.3% and 0.5 ± 0.2%, respectively).

### Cyanobacterial decay

The microbial community structure was analysed *via* high-throughput sequencing targeting the 16S (Bacteria) and 18S (Eukarya) SSU rRNA genes in the samples. The ensuing principal coordinates analysis clustered the community diversity profiles generated according to their non-affected/blighted status (Fig. 3C), supported by ANOSIM statistical tests (p-value < 0.05). Taxonomic assignment of operational taxonomic units (OTUs) (Fig. 3b) revealed that the two types of samples contained significantly different proportions of Cyanobacteria, Bacteroidetes, Stramenopiles, Chloroplastidia, Rhizaria, Alveolata, and Fungi (White’s t-test, p-value < 0.05) (Fig. 3A). The relative abundance of Cyanobacteria was significantly lower in blighted patches, showing an average decrease from 34.1 to 19.5% compared to non-affected areas. Within this phylum, there was a significant reduction of filamentous cyanobacteria (subsection III) attributable to the genus *Leptolyngbya* (Supplementary Fig. 1), which are prevalent mat-forming organisms in polar microbial mats^9^. This reduction in cyanobacterial-related sequences was accompanied by a significant increase (p-value < 0.05) in Bacteroidetes, 30.1% of which were *Flavobacterium*-related and *β-Proteobacteria*-affiliated sequences (Fig. 3A,B and Supplementary Fig. 1).

### Fungal rising

Within Eukaryotes, Stramenopiles (96.4% of which were assigned to Diatomea) comprised the most abundant photo-eukaryotic fraction of the community, and their relative abundance was on average 2.2-fold lower in the blighted communities (Fig. 3A). However, other unicellular eukaryotic groups (including phototrophs) such as Chloroplastidia, Rhizaria and Alveolata had significantly increased relative abundances in blighted patches (Fig. 3A). Fungi-related sequences (mainly *Basidiomycota*-affiliated ribotypes) represented on average 38.4% of the eukaryotic datasets derived from blighted patches (Fig. 3A); however, only 0.8% of the sequences from non-affected patches were assigned to this group (Supplementary Fig. 1). Furthermore, the concentration of ergosterol, a proxy for fungal biomass contribution to total particulate carbon in microbial mats, was on average 25.5-fold higher in blighted patches (2.17 ± 1.78 and 0.09 ± 0.04 mg ergosterol (g dry weight)^−1^, respectively in blighted and non-affected samples); these data were consistent with the significant increase in fungal abundance in blighted patches.

### Viromes divergence

The viral metagenomes from blighted patches predominantly contained several ssDNA viral groups and sequences related to diatom viruses (genus *Bacillarnavirus* and unclassified *Picornavirales*)^10^ (Fig. 3B). However, compared to normal samples, blighted patches contained a higher relative proportion of reads assigned to small DNA and RNA bacteriophages (*Microviridae* and *Leviviridae*, respectively). Notably, the RNA virome from the blighted patches contained sequences assigned to a broad range of eukaryotic viruses; some of these viruses, such as *Partitiviridae* (416 reads) that infect fungi and *Nodaviridae* (928 reads), were not represented in non-affected mats. The emergence of fungal viruses in blighted mats was consistent with the significant increase in fungi detected by 18S rRNA gene sequencing. Notably, BLAST analysis revealed that the partially assembled genomes of nodaviruses were most similar to the highly divergent alphanodavirus found in bat guano viromes^11^. The emergence of small bacteriophages, fungal viruses and rare nodaviruses related to those typically found in saprophyte-dominated environments^12^ might reflect active recycling processes via the “viral shunt”^13^.

## Discussion

Together, these data are consistent with the following scenario. Highly productive microbial mats are dominated by phototropic cyanobacteria and diatoms, and contain the structured and porous physical scaffold required to facilitate efficient transfer of nutrients and metabolites from the physical environment through the foodweb. However, blighted mats have lost important structural properties and have significantly reduced relative abundance levels of the aforementioned phototrophs; instead, blighted mats have increased proportions of copiotrophic heterotrophs such as *Bacteroidetes* and *β-Proteobacteria*^14^. Notably, 18S rRNA gene high-throughput sequencing, measurement of ergosterol concentrations, and nodavirus abundance revealed that the blighted communities have significantly greater numbers of copiotrophic Fungi. Additionally, more abundant putative fungal structures were observed to occupy some of the largest pores in the blighted mat microstructure. Because of this community shift, blighted mats had a denser physical microstructure and greatly decreased proportion of primary producers and consequently lowered N_2_-fixation capability because this process is mostly attributable to diazothrophic cyanobacterial activity in polar microbial mats^15^ in uninterrupted air contact. Notably, decreased total N content and production values mirrored the loss in N-metabolism capabilities.

A feasible explanation for the observed blighting includes a catastrophic breakdown of the cyanobacteria-based ecosystem, enhanced by decomposition and scavenging processes among the saprophytic mat consortia^12^. The initial event triggering progressive decay of the ecosystem remains elusive. We originally proposed that an infectious element might be the blight causative agent, due to the observed damaged patches were reminiscent of *in vitro* viral lysis plaques. However, *in situ* experiments to re-inoculate non-affected mats with ground samples of blighted patches from the same area did not cause blighting over a 3-year period. Furthermore, no evidences for particular causative agents were derived from the metagenomic analyses undertaken. Although cyanobacterial consortia might be affected by viral infection, no significant differences were observed between blighted and non-affected viromes in terms of the viruses known to infect Cyanobacteria. Additionally, lytic bacteria have been related to disruption of the ecosystem for planktonic cyanobacteria^16^. However, blighted patches did not display a significant lytic bacterial profile. Similarly, fungal predation of planktonic cyanobacteria by chytrids is considered an important factor regulating some freshwater ecosystems^17^. Still, the observed increase in fungal populations is associated with *Basidiomycota*-related sequences, while no other fungal taxa significantly varied (Fig. 3B). Notably, we observed putative fungal structures occupying most of the largest pores of the blighted mats. Colonisation of void spaces by those putative *Basidiomycota* hyphae might physically limit the transfer of water and solutes through the mat profile, disturbing biogeochemical gradients^4^. This could trigger a progressive breakdown of the community structure, which is followed by a decay of principal ecological functions, as C and N inputs, and hence impacting over the whole freshwater polar ecosystem.

The presented dataset demonstrate the existence of a seemingly widespread microbial mat blighting-phenomenon in one of the largest ice-free areas and diversity hotspot in maritime Antarctica. Our metagenomic analysis suggests that this phenomenon is not caused by an infective attack on key members of the mat community. A fungal outgrowth might collapse the microstructure of the mat. These Fungi are not truly cold adapted and as a consequence may be disproportionately favoured by warmer temperatures. Increased fungal abundance might have collapse the microstructure and radiate outward over time producing the blighted areas, which is translated into a community structure shift towards a less productive state. The overall results are consistent with *in situ* experiments mimicking global warming in soil communities from the Antarctic Peninsula^18^. Therefore, the blighting phenomenon might be associated with increased local temperatures as Antarctic fungal assemblages are considered not truly cold-adapted organisms^19^ and the Antarctic Peninsula is among the most affected regions by global warming^20^. Given that microbial mats play critical homeostasis role in polar regions, the geographical distribution of the far-reaching blighting events should be investigated in detail.

## Materials and Methods

### Study site and sampling

**Figure 1:**
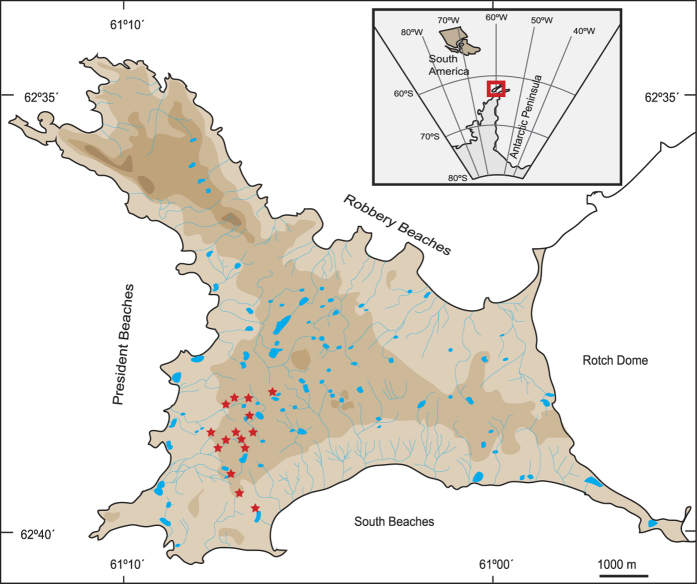
Study-site location. Site map showing the sampling area located in the Byers Peninsula (Livingston Island, South Shetland Islands, Antarctica). Red stars mark surveyed blighted spots. The map is based in the geomorphological map of Byers Peninsula^7^ modified with data from co-authors and shading layers mark 25 m intervals on altitude difference.

**Figure 2:**
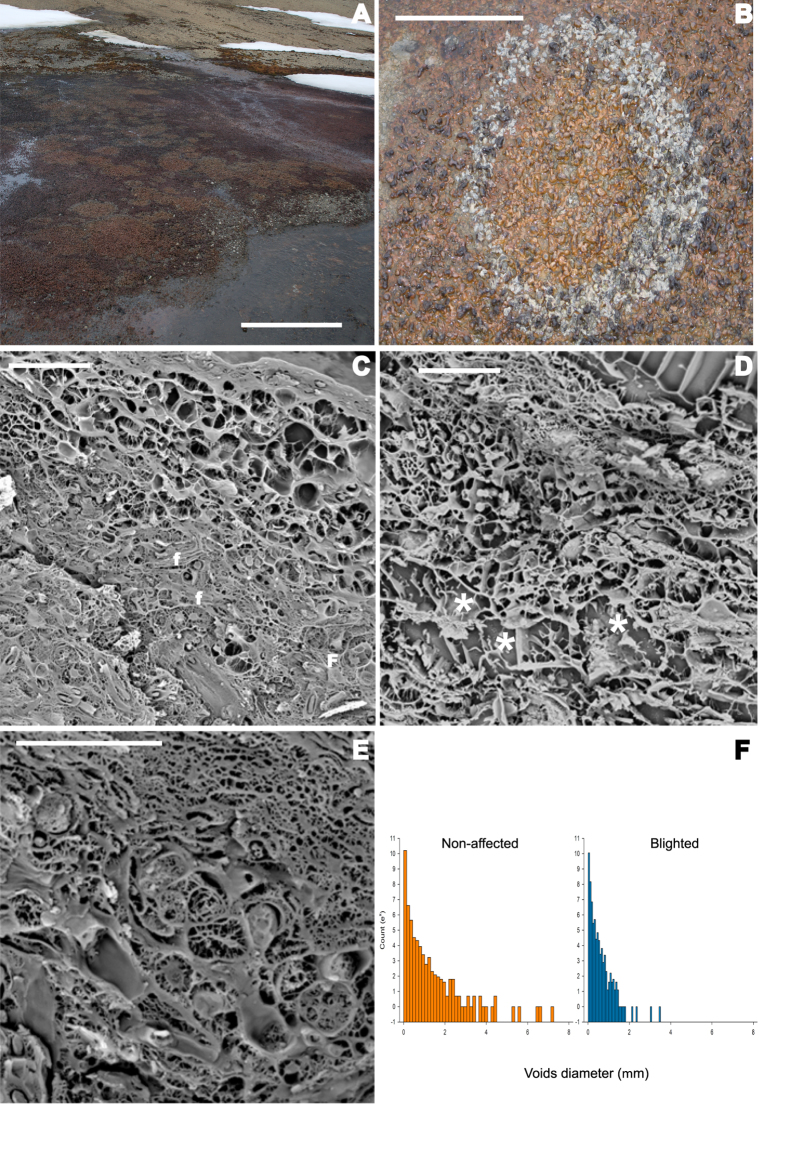
Structural differentiation. **(A**) General view of a microbial mat harbouring several blighted patches. Scale bar, 100 cm. (**B**) Macroscopic features of the blighted patches. Scale bar, 40 cm. (**C–E)** Low Temperature Scanning Electronic Microscopy images of microbial mats from Byers Peninsula. **(C)** Matrix of a microbial mat from blighted patches. **f** indicates putative fungal mycelia within the mat. Scale bar, 20 μm. (**D**) Matrix of a microbial mat from non-affected areas. Asterisks indicate voids with pore diameter greater than 5 μm. Scale bar, 20 μm. (**E**) Fungal mycelia growing in the voids present in blighted microbial mats. Scale bar, 10 μm. (**F**) Histogram representing the pore diameters from non-affected (n = 28,635; 50 bins) and blighted (n = 28,635; 50 bins) samples. Both histograms were generated by analysing images obtained through LTSEM.

**Figure 3:**
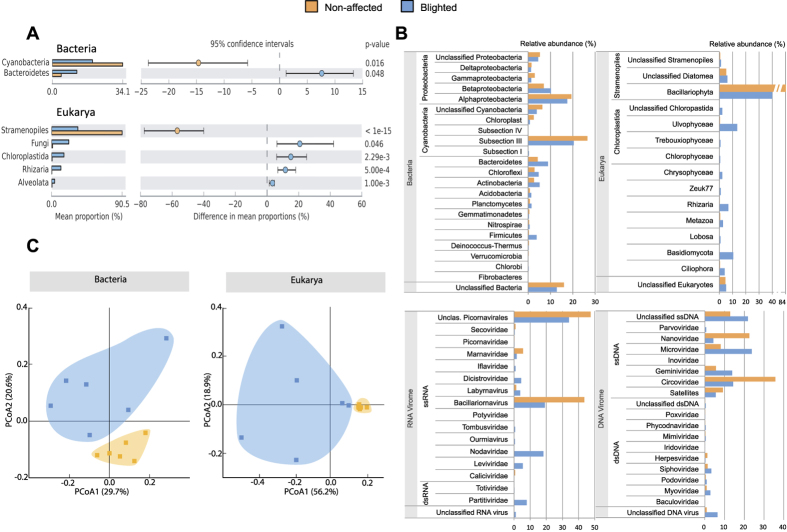
Differences in microbial composition between non-affected and blighted patches. (**A**) Significant differences in the composition of cellular organisms (>95% for the two-sided White’s non-parametric t-test). (**B**) Relative abundance of OTUs from cellular organisms in both types of mats and taxonomic profile (tblastx against viruses database, e value < 0.001) of metagenomic sequences obtained from purified DNA and RNA viral genomes. OTUs were obtained by clustering against the SILVA 111 reference database for 16S and 18S rRNA gene seed sequences and aggregated to the phyla associated with those sequences, except for members of Proteobacteria, Cyanobacteria, Fungi, Stramenopiles and Chloroplastida, which were aggregated to their class when known. For ease of comprehension, the relative abundance was scaled to the total number reads at each respective domain, and only phyla or classes represented over 1% of the relative abundance in each domain are shown. **(C)** Principal component analysis (PCoA) of community structure from cellular organisms explaining 39% of the variability using weighted UniFrac metric of 16S (Bacteria) and 18S rRNA (Eukarya) gene ribotypes.

The study site is Byers Peninsula (Livingston Island, South Shetland Islands, Antarctica —SW Pond; 62°39′10.00′′S, 61°06′21.48′′W), which has a highly developed water network with multiple shallow lakes, ponds, and streams (Fig. 1). The studied community was a multi-layered orange-pigmented microbial mat with a brittle non-uniform surface following the micro-topography of the underlying gravel and an average thickness of 0.7 ± 0.4 cm. The mat grew in a seepage area that almost entirely covered a depression in the landscape. When whitish scattered patches were observed, a complete survey of the area was performed, and all patches were tracked by GPS. A total of 15 main spots were identified at the SW area of Byers Peninsula, where microbial mats were more conspicuous. Most of the spots were composed by a number of blighted patches. Then, nine different blighted patches from different spotted areas and with different diameters were selected for monitoring from 2006 to 2010 by measuring changes in diameter (Supplementary Table 2). Additionally, six samples each from blighted patches and normal-looking areas were used for subsequent analyses.

### Physiological analysis

C and N inputs to the community were measured by isotopic labelled uptake and acetylene reduction assay (ARA), respectively. Inorganic C uptake rates were measured in triplicate from 6 samples of each type. Photosynthetic carbon assimilation was measured as ^13^C (98% ^13^C, Isotec) incorporation in samples after a 2-h incubation^21^. The dissolved inorganic carbon (DIC) content was calculated by considering pH and temperature measured after titration with HCl using a pH shift indicator (phenolphthalein) of equivalence endpoint pH^21^. The total C and N contents were measured from three dried replicates from each type of mat using an elemental analyser with a thermal conductivity detector (Perkin-Elmer 2400CHN).

The N_2_-fixation rates (nitrogenase activity) were also measured through ARA^15^. Briefly, triplicates of six samples were incubated in flasks with acetylene-enriched atmosphere. After 4 h of incubation, ethylene produced by nitrogenase-mediated reduction of acetylene was collected by extracting air from the incubation flasks. Then, the ethylene concentration was determined using a gas chromatograph (Shimadzu GC-8A) equipped with a flame ionisation detector using a Porapak N80/100 column.

### Fugal biomass determination

Ergosterol, a biochemical marker of higher fungal active biomass, was quantified using a HPLC instrument equipped with a UV detector (282 nm). Methanolic extracts of ergosterol were processed in triplicate after refluxing and saponification through a solid phase (SPE) using reverse-phase extraction columns (Waters Sep-Pack Vac RC, 500 mg, tC18)^22^.

### Microscopy analysis

Triplicates samples of hydrated microbial mats from blighted and non-affected mats were cryo-fixed by immersion in liquid nitrogen. The frozen samples were cryo-fractured in the preparation unit, and they were etched for 3 min at −90 °C. After ice sublimation, the etched surfaces were gold sputter-coated, and the samples placed on the cold stage of the SEM chamber. Fractured surfaces were observed using a Zeiss DSM-960 SEM microscope at −135 °C^3^. Equally magnified pictures were used to measure the number and diameter of voids in the samples, and the images were analyzed using ImageJ software combined with the Threshold_Colour plug-in^23^. The resulting data were statistically analysed by ANOVA with Bonferroni post-hoc test when needed.

### Community DNA extraction, SSU RNA amplicon sequencing, and analysis

DNA was extracted with the Power Biofilm DNA isolation kit (MO BIO laboratories, Inc.) according to the manufacturer’s instructions. The bacterial community structure was examined by a bar-coded 16S amplicon sequencing strategy using primers 8F15B (5′-AGAGTTTGATCCTGG-3′) and 515R14AM (5′-TTACCGCGGCTGCT-3′) with specific linking sequences and sequencing adaptors^24^. All PCR reactions were carried out using 1*μ*l of template DNA, 0.5 μl Phusion High-fidelity polymerase (NEB), 20 nmol dNTPs, 20 pmol of each primer, 1.5 *μ*l DMSO, 0.4 mM MgCl_2_, in a final volume of 50 *μ*l. Reaction conditions included an initial denaturation step of 30 s at 98 °C, followed by 25 cycles of 10 s at 98 °C, 30 s at 53 °C, 30s at 72 °C, and a final elongation step of 5 min at 72 °C. For the analysis of the microeukaryotic assemblages we followed a two-step barcoding strategy^24^ that allows a universal set of bar-coded sequencing primers to be appended to an amplified PCR product without introducing discernible biases. In the first step, one of the target-specific primers is modified to include a linker sequence. After amplification, a second primer consisting of the bar code and linker is used to tag the amplicon. The eukaryotic community was analyzed using primers targeting the 18S rRNA gene (F515; 5′-GTGCCAGCMGCCGCGGTAA-3′ and R1119 5′-GGTGCCCTTCCGTCA-3′)^25^. Final concentrations of PCR products were measured using a PicoGreen dsDNA Assay Kit (Life Tech.), equal amounts for each sample pooled, agarose gel-extracted using the QIAquick Gel Extraction Kit (QIAGEN), and sequenced using a Roche 454 GS FLX sequencer with titanium chemistry (Macrogen Inc., Rep. of Korea).

Denoising, filtration of low-quality reads, and removal of chimeras were performed using QIIME 1.6.0^26^. Using the QIIME default settings, reads with mean quality scores below 25, homopolymers runs over 6 nucleotides in length or errors in the primer sequence were discarded. Singletons were discarded to avoid overestimations and clustering bias. Reads were clustered into OTUs with 97% sequence similarity using the uclust_ref algorithm^27^, with rRNA gene sequences from the SILVA rRNA database (release 111)^27^,^28^ used as seed sequences and *de novo* cluster formation allowed. Using this method, a total of 13,443 OTUs were obtained (11,576 for 16S rRNA genes and 1,867 for 18S rRNA genes).

Statistical analyses were performed using Statistical Analyses of Metagenomic Profiles (STAMP) software v 2.0^29^ to detect biologically relevant differences in the relative proportion of classified sequences. PCoA analyses were carried out using QIIME default settings for beta-diversity analysis. The analysis was performed separately for Bacteria and Eukarya profiles from blighted and non-affected samples, and statistical significance was assessed by a two-sided White’s non-parametric t-test^30^. For ease of visualisation, only significant differences (p-value < 0.05) were shown for those taxa that had at least 100 sequences and more than a 2-fold difference between taxa.

### Metagenomic analysis of viruses

Samples were collected from the edge of five blighted patches and from ten different non-affected cyanobacterial mats. Then, 2.5 g from each sample was submerged in 5 ml of SM buffer (Tris-HCl 50 mM pH 7.5, NaCl 100 mM and MgSO_4_.7H_2_O 8 mM) and homogenized by three cycles of vigorous vortexing and sonication in a water bath for 20 and 10 s, respectively, and then centrifuged at 3,000 g for 5 min. This process was repeated twice. The resulting supernatants were combined and centrifuged at 8,000 g for 1 h and then filtered through a 0.45-μm syringe filter (Millex, Durapore PVDF) to remove cellular organisms. The filters were replaced every 2.5 ml to avoid clogging. The resulting viral fractions were pooled by sample type and then concentrated by ultracentrifugation at 60,000 g for 16 h at 4 °C through a 25% sucrose cushion prepared in SM buffer. The samples were maintained at 4 °C during all steps of the procedure to preserve viral integrity. Free contaminating nucleic acids were digested with 500 Uml^−1^ of DNAse I, 100 Uml^−1^ of nuclease S7, 30 μgml^−1^ of RNAse A (Roche) and 10 Uml^−1^ of RNAse H (Invitrogen). After particle disruption with SDS 0.5% and proteinase K treatment (200 μg/ml 45 min 37 °C), the viral metagenomic DNA was obtained by phenol-chloroform extraction, and the viral community RNA was obtained using Trizol-LS (Invitrogen) followed by DNAseI-RNAse free (Roche) treatment. The resulting DNA fraction was amplified using φ29 polymerase and modified random hexamers (GenomiPhi V2, GE Healthcare) for 2 h 10 min at 30 °C. The RNA was first converted to dsDNA and then amplified by sequence-independent single-primer amplification (SISPA)^31^ using 60 pmol of a pseudo-degenerate primer (5′-GTTTCCCAGTCACGATANNNNNNNNN-3′). To prevent external DNA contamination and cross-contamination between samples, all materials were acid-rinsed (0.1 N HCl) and extensively washed with Milli Q water. In addition, all buffers were prepared immediately before use and filtered through 0.22 μm filters. Finally, 1–5 μg of amplified DNA was sequenced using the Roche 454 GS FLX titanium platform (Parque Científico de Madrid, Madrid, Spain). The sequencing output consisted of 102,152 and 22,896 reads for DNA and RNA viromes from blighted mats, respectively, and 329,685 and 31,032 for DNA and RNA viromes from normal cyanobacterial mats, respectively (Supplementary Table 2).

### Quality filtration, assembly and taxonomic binning of viral metagenomic reads

Primer sequences were removed using Biopieces (Hansen, M. A. www.biopieces.org; unpublished), and the PrinSeq suite of tools^32^ was used for quality filtering as previously described^33^. Raw sequences and contigs were assembled using Newbler (minimum 97% identity over a minimum of 90% of the read length). These sequences were compared by BLASTx (E-value < 0.001) with the GenBank nr protein database and by tBLASTx (E-value < 0.001) against a GenBank database with complete viral genomes both on September 2015 (Supplementary Table 3). Every sequence was assigned to taxonomic groups according to the best-hit using MEGAN 4_70_4^34^.

## Additional Information

**How to cite this article**: Velázquez, D. *et al.* Ecosystem function decays by fungal outbreaks in Antarctic microbial mats. *Sci. Rep.* **6**, 22954; doi: 10.1038/srep22954 (2016).

## Supplementary Material

Supplementary Information
