# Supplementary material for: Ecosystem function decays by fungal outbreaks in Antarctic microbial mats
Source: Sci Rep. 2016 Mar 14;6:22954. doi: 10.1038/srep22954 (PMC4789741; doi:10.1038/srep22954)
Supplement: Supplementary Information [file srep22954-s1.doc]

**Ecosystem function decays ­by fungal outbreaks in Antarctic microbial mats** David Velázquez^1^, Alberto López-Bueno^2^, Daniel Aguirre de Cárcer^2^, Asunción de los Ríos^3^, Antonio Alcamí^2^, and Antonio Quesada^1^*.

^1^Departamento de Biología, Universidad Autónoma de Madrid, Madrid, Spain.

^2^Centro de Biología Molecular Severo Ochoa, Consejo Superior de Investigaciones Científicas (CSIC)–Universidad Autónoma de Madrid, Madrid, Spain.

^3^Museo Nacional de Ciencias Naturales–CSIC, Madrid, Spain.

*Correspondence to: [antonio.quesada@uam.es](mailto:antonio.quesada@uam.es)

**Supplementary Materials**

**Supplementary Figure 1. White´s t-test significant differences.** (A) Box-plot analysis of *Leptolyngbya* spp. (p-value=0.017), (B) Basidiomycota (p-value=0.038) and (C) β-Proteobacteria (p-value=0.022). White diamonds indicate the average of each group.

**Supplementary Table 1.** Positioning, and diameter shifts of surveyed blighted round patches from Byers Peninsula (South Shetland Islands, Antarctica).

| Blighted patch code | UTM Coordinates | | Diameter (cm) | |  |
| --- | --- | --- | --- | --- | --- |
|  | Latitude | Longitude | Season |  |  |
|  |  |  | 2006/07 | 2007/08 | 2009/10 |
| #1 | 67º7’45.69’’S | 14º13’35.03’’W | 11x11 | - | 51x43 |
| #2 | 67º7’45.69’’S | 14º13’35.03’’W | 30x30 | 33x35 | 52x50 |
| #3 | 67º7’45.69’’S | 14º13’35.03’’W | 32x32 | 44x39 | 60x55 |
| #4 | 67º7’47.33’’S | 14º13’35.18’’W | 30x30 | 44x45 | 52x45 |
| #5 | 67º7’47.50’’S | 14º13’35.00’’W | 18x18 | 19x21 | 26x21 |
| #6 | 67º7’48.80’’S | 14º13’34.73’’W | 45x45 | 85x70 | 100x80 |
| #7 | 67º7’33.72’’S | 14º13’43.32’’W | 19x20 | 25x30 | 44x34 |
| #8 | 67º7’45.21’’S | 14º13’35.18’’W | 34x34 | 37x40 | 31x30 |
| #9 | 67º7’45.21’’S | 14º13’35.18’’W | 27x27 | - | 50x45 |

**Supplementary Table 2.** Descriptors of viral sequencing data after blast them against GenBank-nr database and GenBank-virus database.

|  |  | **DNA** | | | | **RNA** | | | |
| --- | --- | --- | --- | --- | --- | --- | --- | --- | --- |
|  |  | **Non-affected** | | **Blighted** | | **Non-affected** | | **Blighted** | |
| Reads |  | 329,685 | | 102,152 | | 31,032 | | 22,896 | |
| Average length | | 395 | | 407 | | 309 | | 402 | |
| Primer-clipped reads | |  |  |  |  | 29,972 | | 21,737 | |
| Blast/Dataset | | Blastx-nr | tBlastx-Virus | Blastx -nr | tBlastx -Virus | Blastx -nr | tBlastx -Virus | Blastx -nr | tBlastx -Virus |
|  | Unknown | 296,916 | 297,116 | 89,638 | 89,518 | 22,956 | 24,198 | 13,944 | 17,918 |
|  | Bacteria | 5,089 |  | 1,974 |  | 7 |  | 1,706 |  |
|  | Archaea | 32 |  | 36 |  | 8 |  | 0 |  |
|  | Eukaryota | 1,688 |  | 672 |  | 25 |  | 332 |  |
|  | Viruses | 25,960 | 32,569 | 9,832 | 12,634 | 6,976 | 6,574 | 5,755 | 4,978 |

**Supplementary table 3.** Contigs description of RNA virome from microbial mats from Byers Peninsula (South Shetland Islands, Antarctica)

|  |  |  | blastx |  |  |  |  |  |  |
| --- | --- | --- | --- | --- | --- | --- | --- | --- | --- |
| Contig | Length | Reads | e -value | % identity | Hit | Virus family |  |  | Aligned aa |
| 122 | 699 | 72 | 9.00E-79 | 55 | RNA-dependent RNA polymerase (628aa) | Ustilaginoidea virens partitivirus 2 | | | 121-358 |
| 69 | 877 | 65 | 8.00E-98 | 55 | RNA-dependent RNA polymerase | Fusarium poae virus 1 | |  |  |
|  |  |  |  |  | RNA-dependent RNA polymerase | Ustilaginoidea virens partitivirus 2 | | | 72-221 |
| 127 | 691 | 104 | 1.00E-29 | 35 | Capsid protein (638aa) | Heterobasidion partitivirus 8 | | | 20-236 |
| 168 | 578 | 289 | 1.00E-14 | 41 | Capsid protein (540aa) | Ustilaginoidea virens partitivirus 2 | | | 3-130 |
|  |  |  |  |  | Capsid protein (638aa) | Heterobasidion partitivirus 8 | | | 71-216 |
| 11 | 1,655 | 268 | 5.00E-61 | 33 | putative RdRp | Bat guano associated nodavirus GF-4n | | | 86-540 |
|  |  |  |  |  |  | Pariacoto virus | |  | 95-540 |
| 19 | 1,442 | 324 | 3.00E-41 | 33 | putative RdRp | Bat guano associated nodavirus GF-4n | | | 86-355 |
|  |  |  |  |  |  | Pariacoto virus | |  | 95-368 |
| 130 | 672 | 162 | 5.00E-24 | 34 | NA-dependent RNA polymerase | YT nodavirus | |  | 169-380 |
| 12 | 1,651 | 564 | 6.00E-37 | 30 | coat protein | Alphanodavirus HB-2007/CHN | | | 60-368 |
